# Potent Adjuvanticity of a Pure TLR7-Agonistic Imidazoquinoline Dendrimer

**DOI:** 10.1371/journal.pone.0043612

**Published:** 2012-08-28

**Authors:** Nikunj M. Shukla, Deepak B. Salunke, Rajalakshmi Balakrishna, Cole A. Mutz, Subbalakshmi S. Malladi, Sunil A. David

**Affiliations:** Department of Medicinal Chemistry, University of Kansas, Lawrence, Kansas, United States of America; Centers for Disease Control and Prevention, United States of America

## Abstract

Engagement of toll-like receptors (TLRs) serve to link innate immune responses with adaptive immunity and can be exploited as powerful vaccine adjuvants for eliciting both primary and anamnestic immune responses. TLR7 agonists are highly immunostimulatory without inducing dominant proinflammatory cytokine responses. We synthesized a dendrimeric molecule bearing six units of a potent TLR7/TLR8 dual-agonistic imidazoquinoline to explore if multimerization of TLR7/8 would result in altered activity profiles. A complete loss of TLR8-stimulatory activity with selective retention of the TLR7-agonistic activity was observed in the dendrimer. This was reflected by a complete absence of TLR8-driven proinflammatory cytokine and interferon (IFN)-γ induction in human PBMCs, with preservation of TLR7-driven IFN-α induction. The dendrimer was found to be superior to the imidazoquinoline monomer in inducing high titers of high-affinity antibodies to bovine α-lactalbumin. Additionally, epitope mapping experiments showed that the dendrimer induced immunoreactivity to more contiguous peptide epitopes along the amino acid sequence of the model antigen.

## Introduction

Toll-like receptors (TLRs) are pattern recognition receptors that recognize specific molecular patterns present in molecules that are broadly shared by pathogens, but are structurally distinct from host molecules [1], [2]. The activation of TLRs by their cognate ligands leads to activation of innate immune effector mechanisms, including the production of pro-inflammatory cytokines, and up-regulation of major histocompatibility complex (MHC) molecules and co-stimulatory signals in antigen-presenting cells. The activation of the innate immune system serves to mobilize and amplify subsequent specific adaptive immune responses involving both T- and B-cell effector functions [3]–[6]. Thus, TLR stimuli serve to link innate and adaptive immunity [4] and can therefore be exploited as powerful adjuvants in eliciting both primary and anamnestic immune responses.

At least 10 TLRs are encoded in the human genome [2]. The ligands for these receptors are highly conserved microbial molecules such as lipopolysaccharides (LPS) (recognized by TLR4), lipopeptides (TLR2 in combination with TLR1 or TLR6), flagellin (TLR5), single stranded RNA (TLR7 and TLR8), double stranded RNA (TLR3), CpG motif-containing DNA (recognized by TLR9), and profilin present on uropathogenic bacteria (TLR 11) [7]. TLR1, -2, -4, -5, and -6 respond to extracellular stimuli, while TLR3, -7, -8 and -9 respond to intracytoplasmic pathogen-associated molecular patterns (PAMPs), being associated with the endolysosomal compartment [2].

**Figure 1:**
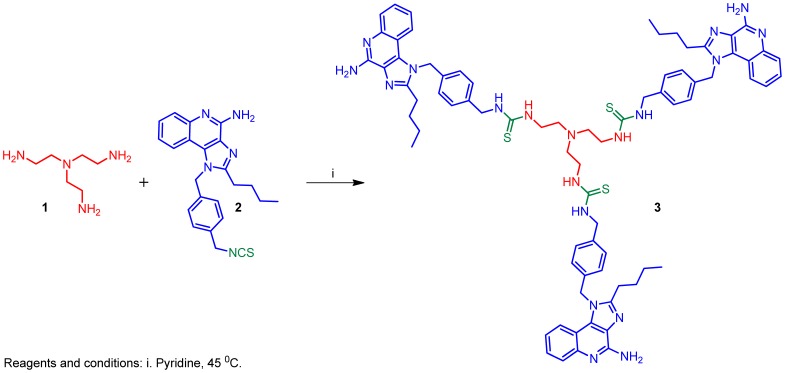
Synthesis of trimeric imidazoquinoline dendrimer 3.

**Figure 2:**
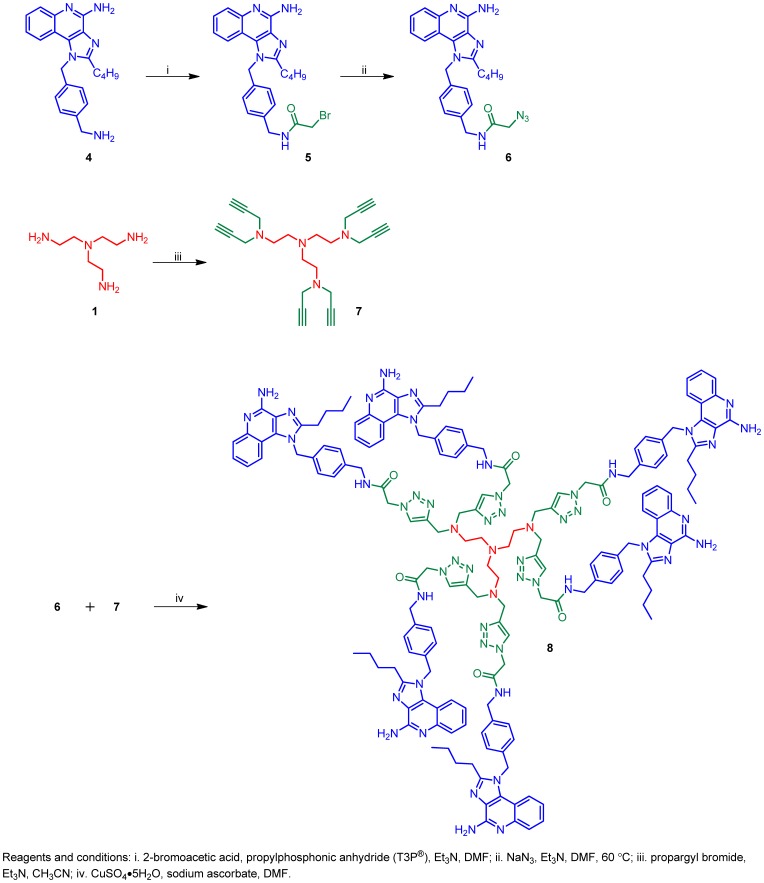
Synthesis of Click reaction derived imidazoquinoline dendrimer 8.

In evaluating representative members of virtually all known TLR agonists in a series of hierarchical assays including primary TLR-reporter assays, secondary indices of immune activation such as cytokine induction and activation of lymphocytic subsets in whole human blood, and tertiary screens characterizing transcriptomal activation patterns with a view to identifying optimal immunostimulatory chemotypes [8], we found that TLR7 agonists, represented by the imidazoquinoline chemotype (Compound **2**, Fig. 1), were extraordinarily immunostimulatory. Extensive structure-activity relationship studies [9]–[13] led to the synthesis of a highly potent TLR7/TLR8 dual-agonistic 1-(4-(aminomethyl)benzyl)-2-butyl-1*H*-imidazo[4,5-*c*]quinolin-4-amine (Compound **4**, Fig. 2), whose free primary amine on the *N* ^1^-benzyl substituent proved a convenient and versatile handle for the attachment of fluorophores [11] as well as a precursor for model self-adjuvanting subunit vaccine constructs with the imidazoquinoline covalently attached to the antigen [13].

Initiation of signaling by TLRs involve homotypic or heterotypic dimerization [14], [15], and we have previously explored the activities of dimeric imidazoquinoline constructs [12]. We asked if a multimeric (dendrimeric) construct of **4** would alter its activity profile. We therefore synthesized a dendrimeric molecule bearing six units of the TLR7/TLR8 dual-agonistic imidazoquinoline **4**. Upon dendrimerization, however, we noted a complete loss of TLR8-stimulatory activity, with selective retention of the TLR7-agonistic activity of its parent monomer in primary screens employing TLR-specific reporter gene assays. This was reflected by a complete absence of TLR8-driven proinflammatory cytokine and interferon (IFN)-γ induction in human PBMCs, but with preservation of TLR7-driven IFN-α induction. The dendrimer was found to be superior to the imidazoquinoline monomer in inducing high titers of high-affinity antibodies. Additionally, epitope mapping experiments showed that the dendrimer induced immunoreactivity to more contiguous peptide epitopes along the amino acid sequence of bovine α-lactalbumin.

## Materials and Methods

All of the solvents and reagents used were obtained commercially and used as such unless noted otherwise. Moisture- or air-sensitive reactions were conducted under nitrogen atmosphere in oven-dried glass apparatus. The solvents were removed under reduced pressure using standard rotary evaporators. Flash column chromatography was carried out using RediSep R_f_ ‘Gold’ high performance silica columns on CombiFlash R_f_ instrument unless otherwise mentioned, while thin-layer chromatography was carried out on silica gel CCM pre-coated aluminum sheets. All intermediates and final target compounds were characterized by ^1^H and ^13^C NMR; spectra were verified to be consistent with their structure. Purity for all final compounds was confirmed to be greater than 97% by LC-MS using a Zorbax Eclipse Plus 4.6 mm × 150 mm, 5 µm analytical reverse phase C_18_ column with H_2_O-isopropanol or H_2_O-CH_3_CN gradients and an Agilent ESI-TOF mass spectrometer (mass accuracy of 10 ppm) operating in the positive ion acquisition mode.

### Synthesis of Compound 3: 1,1',1''-(nitrilotris(ethane-2,1-diyl))*tris*(3-(4-((4-amino-2-butyl-1*H*-imidazo[4,5-*c*]quinolin-1-yl)methyl)benzyl)thiourea)

To a solution of compound **1** (4.2 mg, 0.29 mmol) in pyridine (1 mL) was added compound **2** (46 mg, 1.16 mmol). The reaction mixture was heated at 45°C for 2 h, followed by the addition of polystyrene bound-NH_2_ beads (PS-NH_2_, Biotage) to quench the excess of compound **2**. The reaction was stirred for another 30 min, followed by filtration to remove the beads. The filtrate was concentrated under vacuum and the residue was washed several times with diethyl ether to afford compound **3** (22 mg, 56%). MS (ESI) calculated for C_75_H_87_N_19_S_3_, m/z 1349.65, found 1350.66 (M + H)^+^ and 675.83 (M +2H)^+2^.

**Figure 3:**
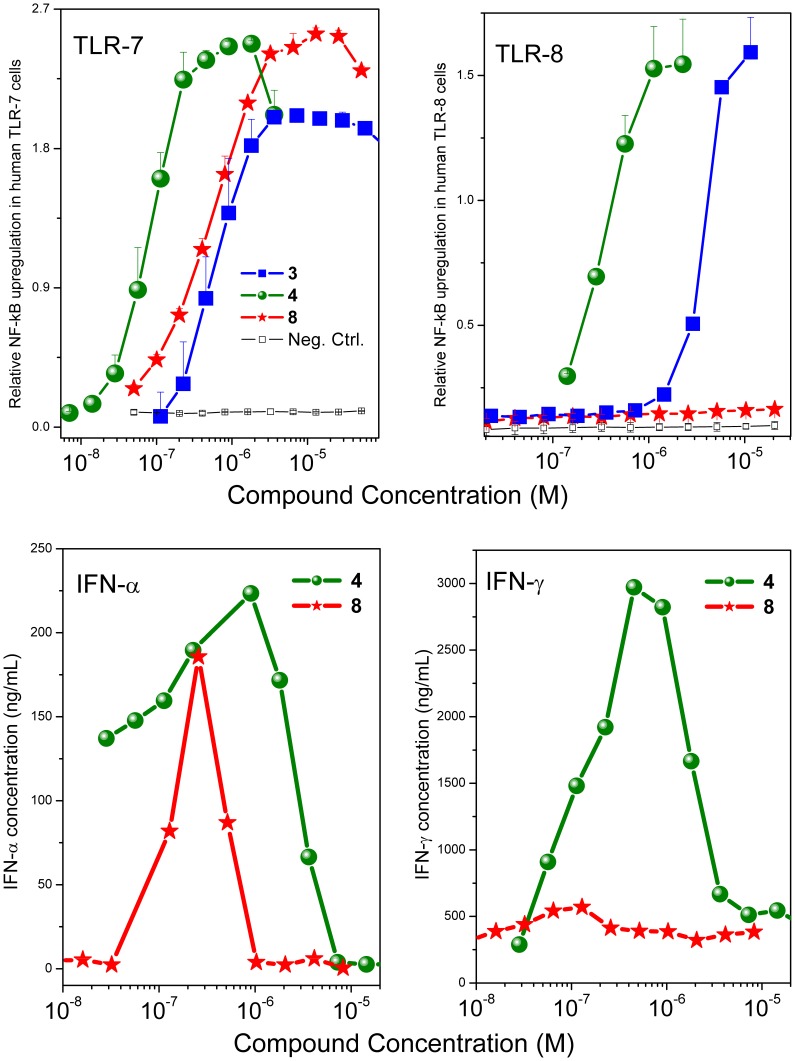
TLR7- and TLR8-agonism and IFN-α and IFN-γ induction by Compounds 3, 4, and 8. Top: TLR7- and TLR8-agonistic activity. Reporter gene assays specific for human TLR7 and TLR8 were used. Data points represent means and SD of quadruplicates. Bottom: IFN-α and IFN-γ induction by Compounds **4** and **8** in human PBMCs, assayed by analyte-specific ELISAs. Data points represent means of duplicates of three representative experiments.

### Synthesis of Compound 6: *N*-(4-((4-amino-2-butyl-1*H*-imidazo[4,5-*c*]quinolin-1-yl)methyl)benzyl)-2-azidoacetamide

To a solution of 1-bromo acetic acid (52 mg, 0.37 mmol) in anhydrous *N*,*N*-dimethylformamide (DMF) were added, triethylamine (130 µL, 0.93 mmol), 50 wt% propylphosphonic anhydride (T3P®) solution in ethylacetate (0.3 mL, 0.48 mmol) and compound **4** (160 mg, 0.37 mmol). The reaction mixture was stirred for 2 h followed by removal of the solvent under vacuum. The residue was then dissolved in ethylacetate and washed thrice with water and brine. The ethylacetate fraction was dried over anhydrous sodium sulfate and then concentrated under vacuum to obtain the crude compound **5** (95 mg). To the solution of compound **5** (95 mg, 0.2 mmol) in anhydrous DMF were added, triethylamine (33 µL, 0.24 mmol) and sodium azide (26 mg, 0.4 mmol). The reaction mixture was then heated at 60°C for 30 min, followed by removal of the solvent under vacuum to obtain the residue which was purified using column chromatography to obtain the compound **6** (55 mg, 34%). ^1^H NMR (500 MHz, MeOD) δ 7.82 (d, *J*  = 8.3 Hz, 1H), 7.66 (d, *J*  = 8.4 Hz, 1H), 7.43 (dd, *J*  = 11.3, 4.1 Hz, 1H), 7.28 (d, *J*  = 8.1 Hz, 2H), 7.12 (t, *J*  = 7.7 Hz, 1H), 7.03 (d, *J*  = 8.1 Hz, 2H), 5.87 (s, 2H), 4.37 (s, 2H), 3.89 (s, 2H), 3.00–2.92 (m, 2H), 1.79 (dt, *J*  = 15.4, 7.6 Hz, 2H), 1.44 (dd, *J*  = 15.0, 7.5 Hz, 2H), 0.93 (t, *J*  = 7.4 Hz, 3H). ^13^C NMR (126 MHz, MeOD) δ 170.15, 156.38, 152.54, 144.50, 139.57, 136.25, 135.70, 129.50, 128.68, 126.92, 125.92, 123.60, 121.68, 115.71, 52.96, 49.62, 43.62, 30.88, 27.84, 23.43, 14.09. MS (ESI) calculated for C_24_H_26_N_8_O, m/z 442.2230, found 443.2345 (M + H)^+^.

**Figure 4:**
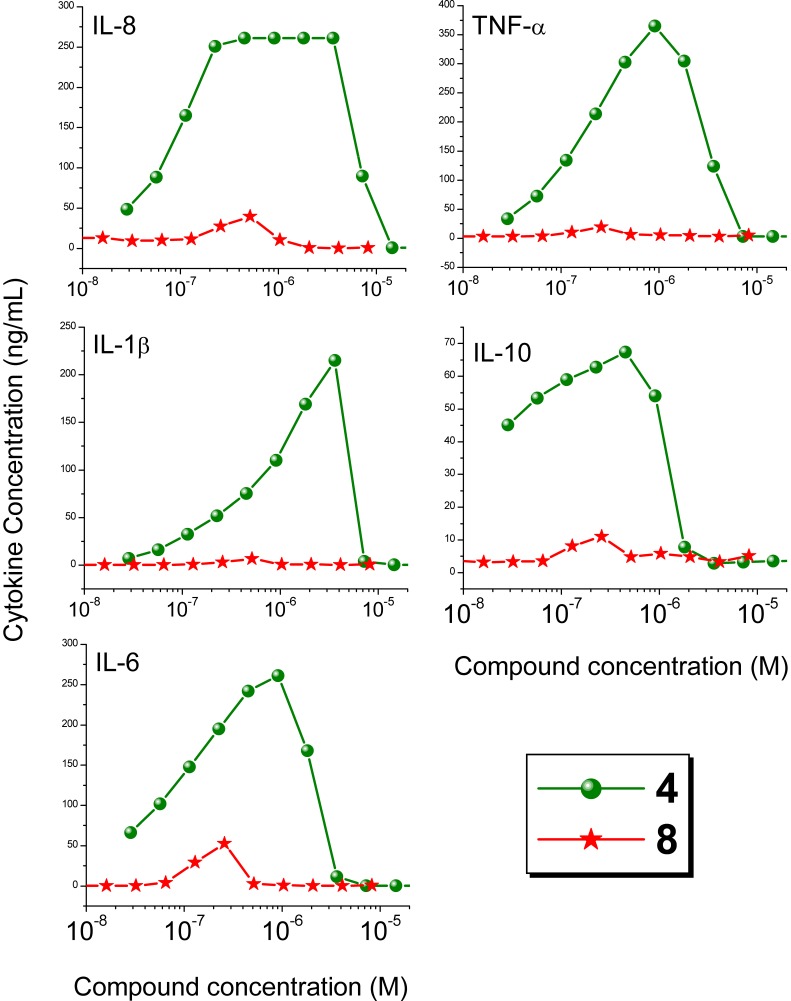
Cytokine induction by Compounds 4 and 8 in human PBMCs. Data points of dose-response profiles represent means of duplicates of three representative experiments. Cytokines were quantified using cytometric bead array assays.

### Synthesis of Compound 7: *N* ^1^,*N* ^1^-bis(2-(di(prop-2-yn-1-yl)amino)ethyl)-*N* ^2^,*N* ^2^-di(prop-2-yn-1-yl)ethane-1,2-diamine

To a solution of compound **1** (299 µL, 2.0 mmol) in CH_3_CN (20 mL) was added triethylamine (1.75 mL, 12.6 mmol). The reaction mixture was cooled to 0°C and propargyl bromide (80% solution in toluene, 2 mL, 13.5 mmol) was added drop wise over a period of 10 min and the reaction mixture was kept stirring at room temperature for 6 h. Water was added to the reaction mixture and the product was extracted in ethyl acetate. The organic layer was washed with water (2×20 mL), brine (2×20 mL) and dried over anhydrous sodium sulfate and concentrated under vacuum. The crude residue was column purified to afford compound **7** as thick liquid (433 mg, 58%). ^1^H NMR (500 MHz, CDCl_3_) δ 3.47 (d, *J*  = 2.4 Hz, 12H), 2.66 (s, 12H), 2.23 (t, *J*  = 2.4 Hz, 6H). ^13^C NMR (126 MHz, CDCl_3_) δ 78.99, 73.23, 52.76, 50.65, 42.71. MS (ESI) calculated for C_24_H_30_N_4_, m/z 374.25, found 375.25 (M + H)^+^.

### Synthesis of Compound 8

To a stirred solution of compound **7** (5.0 mg, 0.013 mmol) and **6** (40 mg, 0.091 mmol) in DMF (2 mL), were added CuSO_4_.5H_2_O (23 mg in 0.5 mL water, 0.091 mmol) and sodium ascorbate (36 mg in 0.5 mL water, 0.18 mmol) and the reaction mixture was stirred at room temperature for 1 h. The dendrimer formed was purified by semi-preparative reverse phase HPLC to afford compound **8** as solid (15 mg, 38%). MS (ESI) calculated for C_168_H_186_N_52_O_6_, m/z 3027.5848, found 1514.8233 (M +2H)^+2^ and 1010.8771 (M +3H)^3+^.

**Figure 5:**
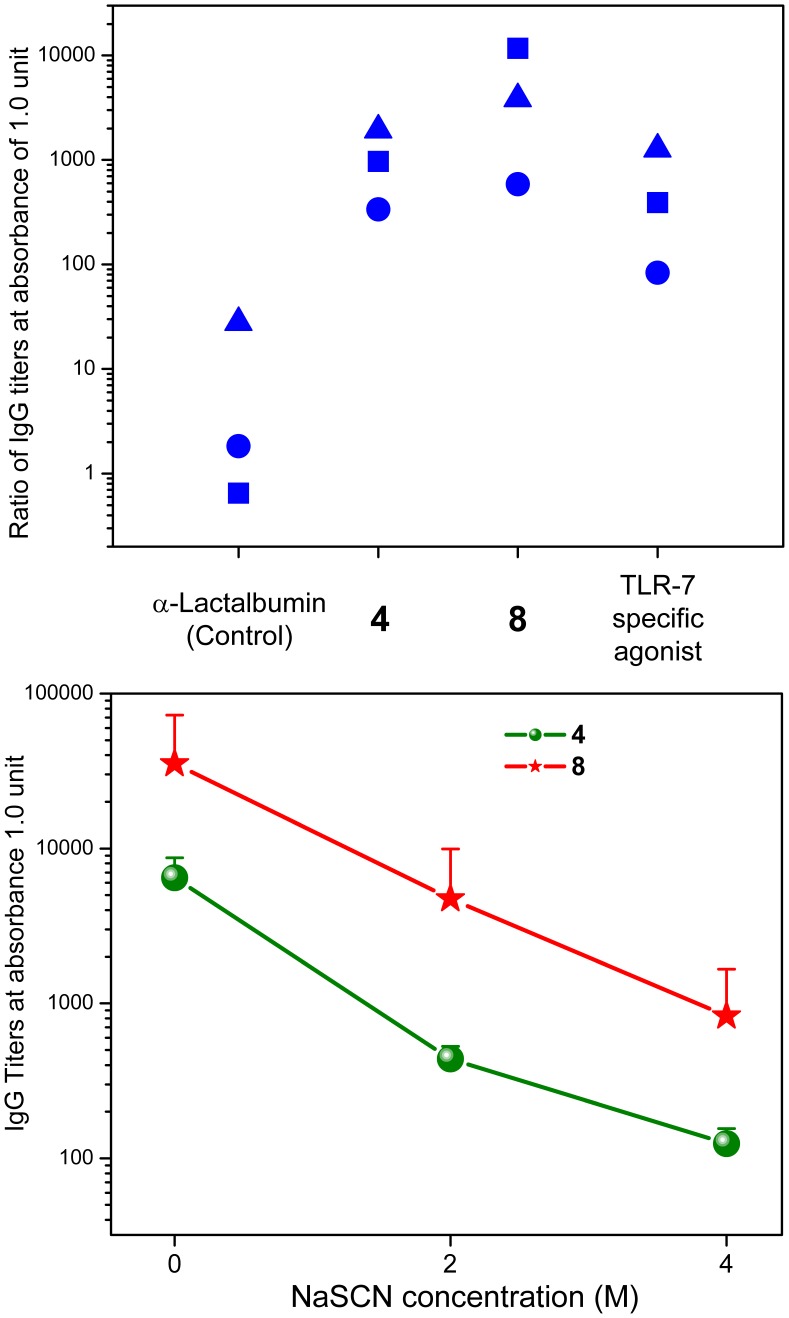
Anti-bovine α-lactalbumin-specific IgG titers. Top: Anti-bovine α-lactalbumin-specific IgG titers in rabbits adjuvanted with **4**, **8**, a TLR7-specific imidazoquinoline (1-benzyl-2-butyl-1*H*-imidazo[4,5-*c*]quinolin-4-amine; reported as Compound 31 in Ref. 9), and unadjuvanted controls (n = 3 per cohort). Ratios of immune/pre-immune titers yielding absorbance values of 1.0 are shown for the individual samples. Each symbol corresponds to the titer of a single animal. Bottom: Chaotropic ELISA showing apparent titers as a function of chaotrope (NaSCN) concentration. Means and SD are shown.

### TLR7 and TLR8-specific Reporter Gene Assays

The induction of nuclear factor-kappa B (NF-κB) was quantified using Human Embryonic Kidney (HEK)-Blue-7 cells and HEK-Blue-8 cells stably expressing human TLR7 and human TLR8, respectively, as previously described by us [8], [9].

**Figure 6:**
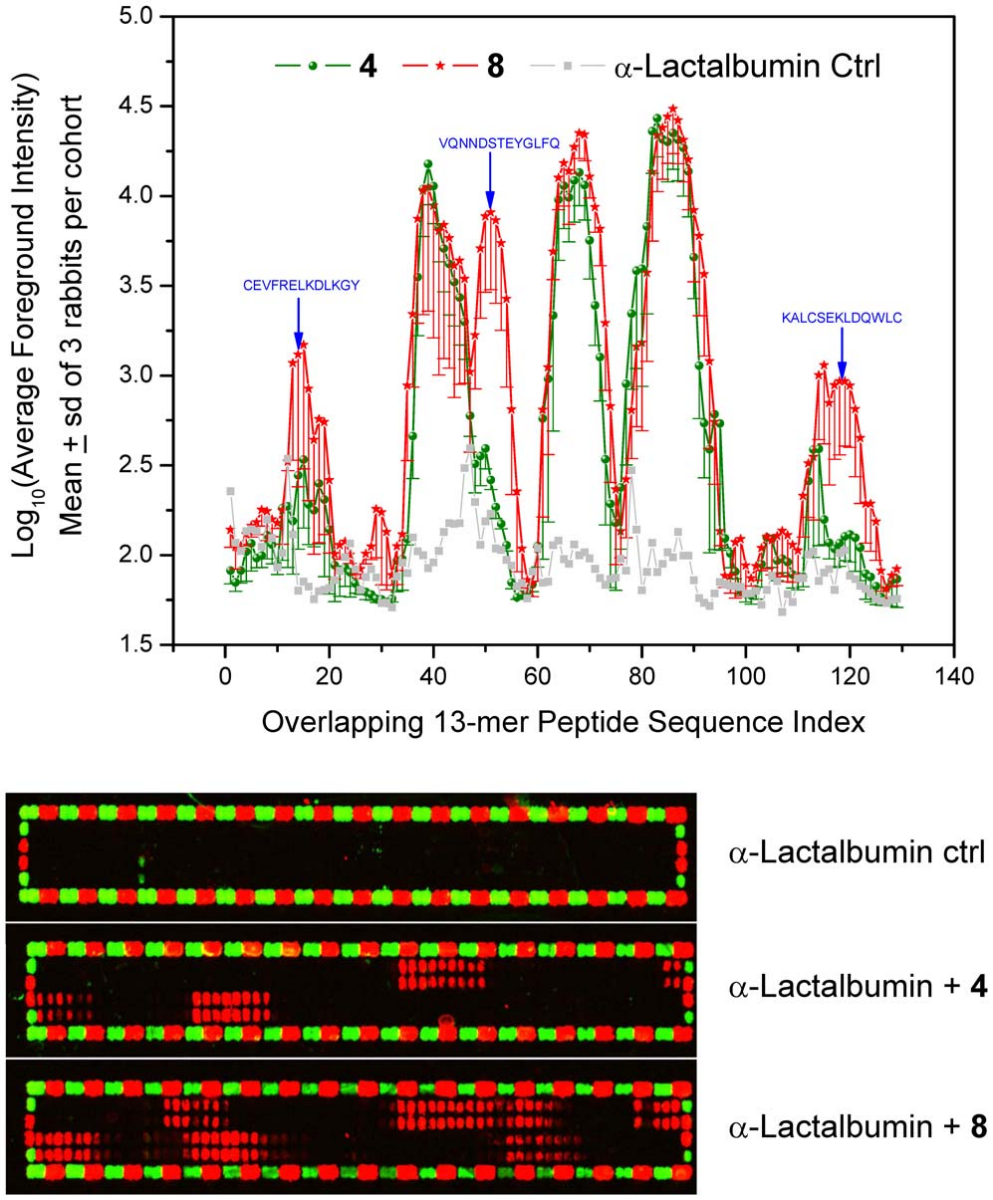
Linear peptide epitope mapping results of immune sera. Top: Foreground fluoresence intensities of secondary goat anti-rabbit IgG (H+L) conjugated with DyLight680 antibody to each overlapping 13-mer peptide in the microarray are shown (means and SD). Bottom: Raw fluorescence images of representative peptide microarrays are shown. Each array is framed by a fusion tag (Flag) peptide (DYKDDDDKGG, 72 red spots) and influenza virus hemagglutinin (HA) epitope tag peptide (YPYDVPDYAG, 72 green spots) which were used as internal controls.

### Assays for IFN-α, IFN-γ, and Cytokines

Fresh human peripheral blood mononuclear cells (PBMCs) were isolated from human blood obtained by venipuncture using conventional Ficoll-Hypaque gradients as described elsewhere [12]. Venipuncture was performed with informed, written consent on healthy adult males with no acute illnesses. Venipuncture procedures were approved by the University of Kansas Human Subjects Committee (KU-HSCL Approval # 12397). Aliquots of PBMCs (10^5^ cells in 100 µL/well) were stimulated for 12 h with graded concentrations of test compounds. Supernatants were isolated by centrifugation, diluted 1∶20, and were assayed in triplicates using a high sensitivity analyte-specific enzyme-linked immunosorbent assays (ELISA) kits (PBL Interferon Source, Piscataway, NJ and R&D Systems, Inc., Minneapolis, MN). Cytokine production was examined using a FACSArray multiplexed flow-cytometric bead array (CBA) system (Becton-Dickinson-Pharmingen, San Jose, CA) as described by us previously [16].

**Figure 7:**
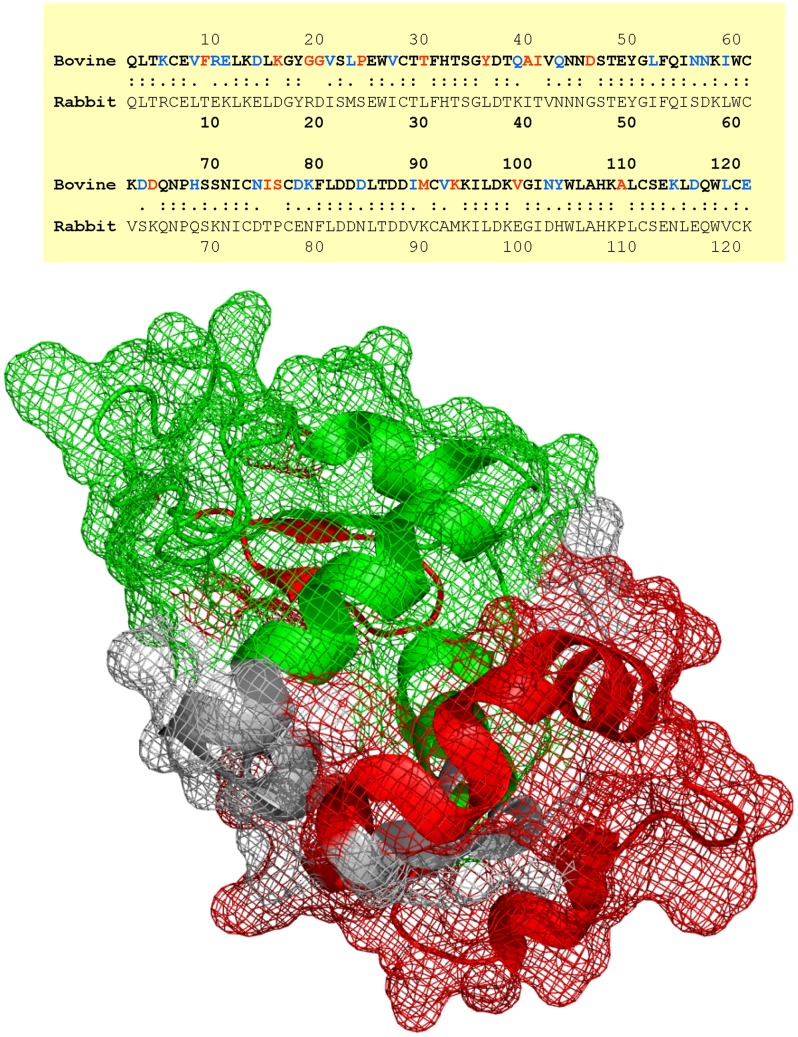
Mapping of immunoreactive linear epitopes of bovine α-lactalbumin to its primary sequence and crystal structure. Top: Sequence homology between bovine and rabbit α-lactalbumin. Conservative and non-conservative changes are shown in blue and red, respectively. Bottom: Mapping of immunoreactive linear epitopes to the crystal structure of bovine α-lactalbumin (PDB code: 1F6R). Regions colored green show contiguous immunodominant epitopes elicited by both **4** and **8**, while regions colored red show additional epitopes specifically adjuvanted by **8**.

### Rabbit Immunization

All experiments were performed at Harlan Laboratories (Indianapolis, IN) in accordance with institutional guidelines (University of Kansas IACUC permit # 119-06) which specifically approved this study. All antigen/adjuvant preparations were entirely aqueous; no liposomal or emulsifying agents were used. Cohorts of adult female New Zealand White rabbits (n = 3) were immunized intramuscularly in the flank region with (a) 100 µg of bovine α-lactalbumin in 0.2 mL saline, or (b) 100 µg of bovine α-lactalbumin plus 100 µg of **4** in 0.2 mL saline, or (c) 100 µg of bovine α-lactalbumin plus 100 µg of **8** in 0.2 mL saline, or (d) 100 µg of bovine α-lactalbumin plus 100 µg of a high-potency, pure TLR7-agonistic imidazoquinoline (1-benzyl-2-butyl-1*H*-imidazo[4,5-*c*]quinolin-4-amine; reported as Compound **31** in Ref. 9). Pre-immune test-bleeds were first obtained via venipuncture of the marginal vein of the ear. Animals were immunized on Days 1, 15 and 28. A final test-bleed was performed via the marginal vein of the ear on Day 38. Sera were stored at −80°C until used.

**Figure 8:**
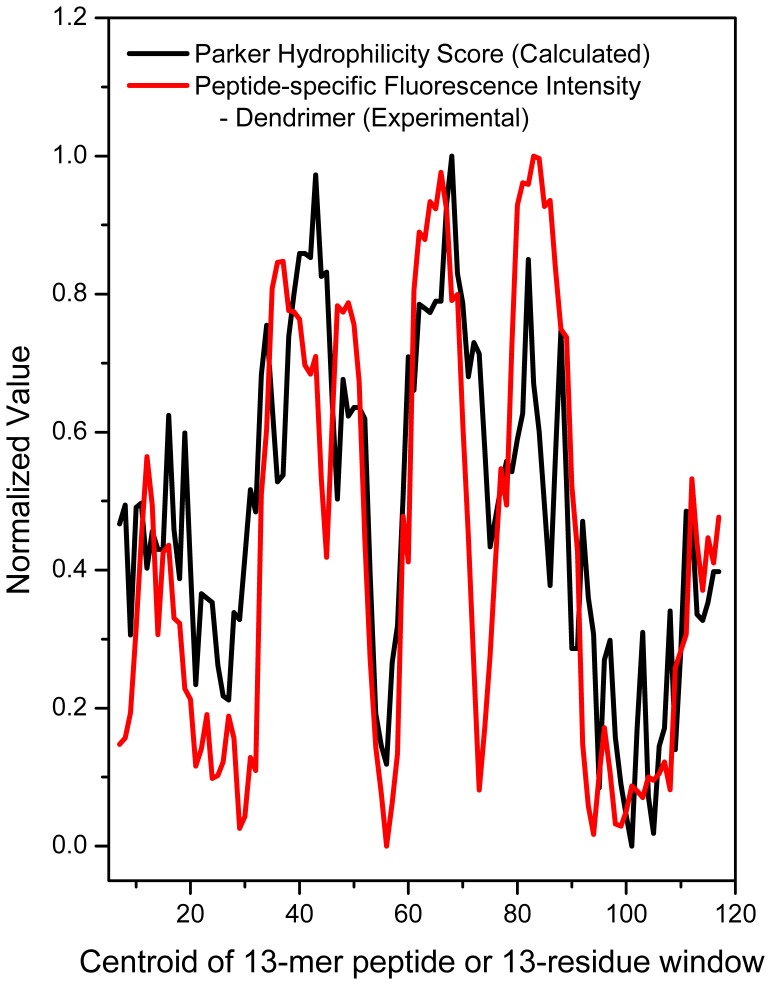
Correlation of observed and predicted epitopes. Overlay of normalized mean background-corrected fluorescence intensity (13-mer peptide-specific immunoreactivity) of the dendrimer **8-**adjuvanted rabbit sera and normalized Parker Hydrophilicity parameter computed with a moving window of 13 residues.

### Enzyme-linked Immunosorbent Assays (ELISA)

Bovine α-lactalbumin-specific ELISAs were performed in 384-well format using automated liquid handling methods as described by us [13]. Examination of the affinity of antigen-specific IgG using chaotropic ELISA [17], [18]. A precision 2000 liquid handler (Bio-Tek, Winooski, VT) was used for all serial dilution and reagent addition steps, and a Bio-Tek ELx405 384-well plate washer was employed for plate washes; 100 mM phosphate-buffered saline (PBS) pH 7.4, containing 0.1% Tween-20 was used as wash buffer. Nunc-Immuno MaxiSorp (384-well) plates were coated with 30 mL of α-lactalbumin in 100 mM carbonate buffer, pH 9.0 overnight at 4°C. After 3 washes, the plates were blocked with 3% bovine serum albumin (in PBS, pH 7.4) for 1 h at rt. Serum samples (in quadruplicate) were serially diluted in a separate 384-well plate using the liquid handler. After three additional washes of the assay plate, 30 µL of the serum dilutions were transferred using the liquid handler, and the plate incubated at 37°C for 2 h in the absence or presence of graded (2M and 4M) concentrations of sodium thiocyanate. The assay plate was washed three times, and 30 µl of 1∶10,000 diluted appropriate anti-mouse immunoglobulin (IgG [γ chain], IgM [µ chain], IgG1, IgG2a) conjugated with horseradish peroxidase was added to all wells. Following an incubation step at 37°C for 1 h, and three washes, tetramethylbenzidine substrate was added at concentrations recommended by vendor (Sigma). The chromogenic reaction was terminated at 30 min by the addition of 2M H_2_SO_4_. Plates were then read at 450 nm using a SpectraMax M4 device (Molecular Devices, Sunnyvale, CA).

### Linear Epitope Mapping (ELISA)

Linear peptide epitope mapping were performed utilizing PEPperMAP® technology (PEPperPRINT GmbH, Heidelberg, Germany). Immune sera (Day 38) from three animals in each cohort were used. The C- and N-termini of the bovine α-lactalbumin were first elongated by neutral GSGSGSG sequences to avoid truncated peptides. The protein sequence was then translated into 13-mer peptides with a peptide-peptide overlap of 12 amino acids. Arrays of 129 peptides were printed in duplicate spots; four such arrays were printed on each glass slide. Each array was framed by a fusion tag (Flag) peptide (DYKDDDDKGG, 72 spots) and influenza virus hemagglutinin (HA) epitope tag peptide (YPYDVPDYAG, 72 spots) as controls. After pre-swelling the arrays for 10 min in standard buffer (phosphate-buffered saline [PBS], pH 7.4+0.05% Tween 20) and 60 min in Rockland blocking buffer (Rockland Immunochemicals, Inc., Gilbertsville, PA), the peptide microarrays were initially incubated with the secondary goat anti-rabbit IgG (H+L) conjugated with DyLight680 antibody at a dilution of 1∶5000 for 60 min at room temperature to verify that no significant background interactions occurred with the peptide arrays. The microarrays were washed twice, and incubated for an additional 30 min in standard buffer. The peptide arrays were then incubated overnight at 4°C with rabbit sera diluted to 1∶1000. After multiple washes in standard buffer, the slides were incubated for 30 min with the secondary goat anti-rabbit IgG (H+L) conjugated with DyLight680 antibody at a dilution of 1∶5000 at room temperature. After two additional washes in standard buffer, the microarrays were rinsed with ultrapure water and dried in a stream of air. Green/red fluorescence intensities were acquired on an Odyssey Imager (Lincoln, NE) at a spatial resolution of 21 µm. Staining of Flag and HA control peptides that frame the arrays gave rise to high and homogeneous spot intensities with a coefficient of variation of <2%. The PEPSlide Analyzer algorithm deconvolutes raw fluorescence intensities of each spot into foreground and background signal. Intensity maps were generated based on corrected foreground intensities (averaged over the double spots) of each peptide.

## Results and Discussion

As mentioned earlier, we were desirous in examining dendrimeric constructs of the imidazoquinoline with to view to testing the hypothesis that such larger constructs may enhance adjuvanticity. We did not, however, know *a priori* if such molecules would even retain TLR7/8 agonistic activity. Our first attempt at evaluating such dendrimeric molecules began with the synthesis of **3** (Fig. 1) which was accomplished by reacting an isothiocyanate derivative **2** that we had used earlier [11], [13] with the triamine **1**. We were gratified that this trimeric molecule **3** retained, in large measure, both the TLR7- and TLR8-agonistic activities of the parent monomeric imidazoquinoline (Fig. 1). We next extended our efforts in synthesizing a hexameric construct **8**, which was obtained via a ‘click’ reaction of the azide-bearing **6** with the hexa-alkyne **7** (Fig. 2). To our surprise, we noted that while the dendrimer **8** retained substantial TLR7-agonistic activity, its TLR8-stimulating properties appeared entirely abrogated in primary screens employing reporter gene assays (Fig. 3).

In recognition that bioactivity readouts using cell-culture systems may not always reflect with fidelity in vivo behavior owing to a variety of reasons, including differential plasma protein binding (that we ourselves have observed and characterized) [19], it was important to verify that the activity profiles observed in TLR-specific reporter gene assays was also seen in primary human cells. It is to be noted that we [10], [16], [20] and others [21]–[23] have shown that pure TLR7 agonists selectively induce Type I interferons, while the production of IFN-γ and proinflammatory cytokines is attributable primarily to TLR8 activation. We confirmed that the dendrimer was selectively bereft of TLR8-agonistic properties in secondary screens using human PBMCs in which the dendrimer **8**, unlike its parent monomer **4**, showed selective induction of TLR7-driven IFN-α, but not of IFN-γ (Fig. 3), or proinflammatory cytokines such as TNF-α, IL-1β, and IL-6 (Fig. 4).

It is to be noted that both in interferon (Fig. 3) and in cytokine-release assays (Fig. 4), a bimodal dose-response profile characterized by an initial dose-dependent increase in analyte concentration followed by an apparent suppression in interferon/cytokine production was observed as we have previously reported [10], [13], [16]. The origin and cause of this bimodal response is not clear, but we verified that this is not due to cytotoxicity using standard XTT [24] and resazurin [25] assays.

Immunization of rabbits with bovine α-lactalbumin with either the monomeric compound **4** or the dendrimer **8** elicited strong humoral responses (Fig. 5); however, although not statistically significant owing to the small sample sizes, anti-α-lactalbumin IgG titers in rabbits adjuvanted with **8** were higher than titers elicited by **4**, or a high-potency, pure TLR7-agonistic imidazoquinoline (1-benzyl-2-butyl-1*H*-imidazo[4,5-*c*]quinolin-4-amine; reported as Compound **31** in Ref. 9**)** (Fig. 5). Affinity measures of antibody quality using chaotropic ELISAs also indicated that the use of **8** as adjuvant resulted in significantly higher-affinity antibodies (Fig. 5). Linear epitope mapping clearly showed that the dendrimer **8** induced immunoreactivity to more contiguous peptide epitopes along the amino acid sequence of bovine α-lactalbumin. First, a pronounced immunoreactivity to a peptide sequence centered around amino acids 42–54 (VQNNDSTEYGLFQ) was observed in animals adjuvanted with **8**; second, higher immunoreactivity to peptide sequences centered on amino acids 6–18 (CEVFRELKDLKGY) and 108–120 (KALCSEKLDQWLC) were also observed (Fig. 6). These data clearly show that immunization with **8** as adjuvant extends the immunoreactivity profile of the humoral response to encompass longer stretches of the amino acid sequence of the protein antigen. These results were somewhat unexpected because an examination of the amino acid sequences of bovine and rabbit α-lactalbumin shows differences throughput the length of the protein (Fig. 7; Waterman-Eggert score: 579; 61.2% identity; 85.1% similar), and we had surmised, perhaps naïvely, that the immunoreactivity patterns of linear epitopes in the monomer- and dendrimer-adjuvanted samples would be very similar, with the dendrimer evoking stronger responses. The mechanism(s) underlying the superior adjuvanticity of **8** are unknown; we hypothesize that this may be related to the lack of induction of counter-regulatory IL-10.

The availability of experimentally determined linear epitope mapping data provided an opportunity to test and benchmark known linear epitope prediction algorithms [26]. We compared six methods that are implemented in Immune Epitope Database Analysis Resource (www.immuneepitope.org): (a) Chou-Fasman β-turn prediction propensity [27]; (b) Emini surface accessibility parameter [28]; (c) Karplus and Schulz flexibility prediction [29]; (d) Kolaskar and Tongaonkar method [30]; (e) Parker hydrophilicity prediction [31]; (f) Bepipred linear epitope prediction method [32]. The Parker hydrophilicity predicition algorithm yielded a profile that correlated best with the experimentally-determined linear epitope immunoreactivity patterns (Fig. 8).

It would also be pertinent to note two additional points: First, the absolute concentration of 100 µg/dose of **4** and **8** are, respectively, 278 nMoles and 33 nMoles; therefore, lower concentrations of **8** exhibits higher adjuvantic activity than **4**, inducing higher titers of antibodies, which are also of higher quality as adjudged by chaotropic ELISA and epitope mapping. Second, although no apparent local or systemic adverse effects were reported for any of the rabbits, the dendrimer **8**, unlike **4**, is a pure TLR7 agonist, inducing only Type I IFN, and no proinflammatory cytokines. Based on current paradigms, one would predict far lower reactogenicity for **8**. Accordingly, a detailed comparison of local and systemic reactogenicity with biomarker profiling is being planned.

### Ethics Statement

All immunization experiments involving animals were performed at Harlan Laboratories (Indianapolis, IN) in accordance with institutional guidelines (University of Kansas IACUC permit # 119-06) which specifically approved this study. Venipuncture was performed with informed, written consent on healthy adult males with no acute illnesses. Venipuncture procedures were approved by the University of Kansas Human Subjects Committee (KU-HSCL Approval # 12397).
